# Revision of hospital work organization using nurse and healthcare assistant workload indicators as decision aid tools

**DOI:** 10.1186/s12913-019-4376-7

**Published:** 2019-08-07

**Authors:** Isabelle Briatte, Caroline Allix-Béguec, Gérard Garnier, Mercédès Michel

**Affiliations:** 1 Coordination générale des soins, Groupe Hospitalier de la Rochelle Ré Aunis, CH La Rochelle, rue du Dr Schweitzer, 17019 La Rochelle, France; 2 Unité de recherche clinique, Groupe Hospitalier de la Rochelle Ré Aunis, CH La Rochelle, rue du Dr Schweitzer, 17019 La Rochelle, France

**Keywords:** Resource management, Information management, Workforce planning, Change process, Nurses, Healthcare

## Abstract

**Background:**

Historically, governmental hospital organisation consisted in a heterogeneous distribution of staff and a fragmented logistical organisation without cross-functionality or sharing of resources between departments. This organisation could not last in a context of an evolving healthcare environment, changing patient profiles and hospital expenditure constraints. Cost-effective workforce regulation for optimal patient quality of care was urgently needed. The purpose of the study was to describe the reorganization that led to resource management no longer based on what has been achieved but based on a daily measured workload.

**Methods:**

This prospective study used nursing intensity indicator, mirroring patient care needs, which was reported daily using VALPAReSO® software. Indirect care activities were recorded in departments of medicine, surgery and obstetrics. Based on data collected in 2012, a new organisation strategy was implemented and evaluated in 2015.

**Results:**

Nursing intensity indicator analysis led to a reallocation of workforce per department, and the reinforcement unit (float pool) was managed based on this decision-aid tool for replacement and daily adequate staffing. The healthcare workflow audit resulted in the revision of five working tasks: time spent on handover, working time management, connections between services and the pharmacy, housekeeping, and food management. The reorganization took place at the same time as the transition to the development of very short-term care, resulting in a decrease in the number of full inpatient beds, which were therefore mainly occupied by heavier care profile patients. With the integrated strategy, this transition was achieved with constant staffing, and good overall patient satisfaction and working conditions were maintained.

**Conclusion:**

The reorganisation strategy was managed in a context of institutional commitment, coaching leadership built on close manager-employee interaction, a defragmented management between healthcare and all service providers, and a seamlessly dissemination and sharing of indicator information between healthcare managers, nurses and healthcare assistants. The process optimization allowed a better allocation of tasks and enabled nurses to refocus on patient care. Nursing intensity and indirect care indicators, when widely accepted, can be used as decision support tools for daily adequate staffing.

**Electronic supplementary material:**

The online version of this article (10.1186/s12913-019-4376-7) contains supplementary material, which is available to authorized users.

## Background

To enable continuous improvement of quality of care in health facilities, tools and methods are required to provide knowledge of health systems. Diagnosis-related group is a classification system that categorize patients with respect to diagnosis, treatment and length of hospital stay [1]. It was introduced to better describe hospital services and to improve measurement and management of hospital services. It provides indicators on medical activities and management costs of different pathologies, but it does not link patient needs with healthcare provider workload. Therefore, additional tools are necessary to quantitatively and qualitatively assess the daily activities of nurses and healthcare assistants, and to provide decision support system in term of work organization, working condition improvement, and adequate staffing according to patient needs.

The workload indicators had to be useful, reliable, and recognized by professionals as being representative of the paramedic activity in accordance with patient actual needs, while being accepted by the medical and administrative bodies. Several measurement tools of nursing intensity exist like the PRN in Canada used since the 70^s^ [2], the RAFAELA™ system owned by the Association of Finnish Local and Regional Authorities [3], the Safer Nursing Care Tool, a NICE endorsed tool [4], the Scottish Community Nursing Workload Measurement Tool [5] and the Pendiscan [6], a French system that determines patient dependency profiles. In France, the most widely used method is called SIIPS® [7]. It provides a common methodology for measuring the burden of care and for obtaining a model estimating the workload of hospital care through explanatory variables. In this model, nursing intensity covers direct patient care including basic care (feeding, waste disposal, hygiene, dressing, comfort and locomotion), technical care (diagnostic and therapeutic procedures consecutive to medical prescription) and relational care (information and support to patients and families). A study of the computerized version demonstrated the feasibility, the liability of this indicator, and the compliance of nurses to the system [8]. Nursing intensity does not measure the whole nursing service as some care-related activities are not taken into account. Nurses and healthcare worker assistants also ensure tasks that do not directly affect the patient. These tasks correspond mainly to administrative tasks, logistics, phone, training, and management. They are directly related to institution and health department organisations. A specific methodology was designed and validated by the French Ministry of social affairs, health and city in order to standardized the measurement of indirect care in French healthcare institutions [9].

Our governmental hospital organization historically relied on a heterogeneous distribution of staff resources per department, without this inequity ever being measured. There were units known for their “heavy” workloads and others recognized as “lighter”. The nursing model of care was a total patient care model. The logistics organization was built on a segmented model without shared means between the different providers. In each department, the logistical tasks were provided by the department’s caregivers. Paramedical human resources (nurses, physiotherapists, psychomotors, speech therapists, and radiographers), healthcare assistants and cleaners participated in transport and storage of equipment. Depending on the needs of the moment and availability, they could collect drugs from the pharmacy, evacuate waste to specialized premises, and collect specific food in the kitchen… In a context that was becoming more and more tightly controlled for all healthcare institutions, we had to face the need to adjust our organisations and make them more efficient. The subjectivity of workload needed to be objectified in order to optimize paramedical human resources of care units. An audit of our logistic organizations was necessary in order to determine all that could be entrusted to logistic professionals (couriers, storemen, coordinators), and therefore relieve nurses and other caregivers from these tasks not directly related to patient care. In the context of an evolving healthcare environment, changing patient profiles and hospital expenditure constraints [10], it was essential to regulate the workforce in a cost-effective way while ensuring optimal patient quality of care.

The aim of this study was to implement healthcare workload indicators as decision support tools in order to audit the logistics of the organisation, so that the distribution of resources was no longer based on what has been achieved but on a measured, objective workload on a daily basis and according to the fluctuation in patient care. This healthcare productivity model aims at being flexible and happening in real time unlike the previous model which was based on past data analysis.

## Methods

### Setting

The study was held in a government hospital providing healthcare in medicine, surgery, obstetrics, geriatrics and psychiatry. Due to the merger of several health facilities, its capacity increased from 1679 to 1951 beds in 2011 and 2015, respectively. The number of full-time equivalent of healthcare workers increased from 3429 in 2011 to 3716 in 2015. The average bed occupancy level was over 85%, and more than 215,000 hospital days were recorded per year.

### Study population

Nurses, healthcare assistants and cleaners, from 23 departments in 2012 that were merged into 20 departments in 2015 (gynaecology, neonatology, paediatrics, cardiac intensive care, cardiology, diabetes, inpatient cardiology, internal medicine, medicine, rheumatology, gastroenterology, orthopaedics, urology, vascular and thoracic surgery, visceral surgery, neurology, oncology, pulmonary department, intensive care unit, short term geriatric assessment), participated in the study. They were asked to provide data on nursing intensity and indirect care.

### Study design

This was a prospective observational study based on healthcare provider workload recordings in 23 departments from the same institution. It was a descriptive study on nursing intensity. Results were compared for indirect care, patient and healthcare worker satisfaction surveys between 2012 and 2015.

### Staff workload indicators

Nursing intensity (SIIPS score) gave an overall and synthetic assessment of daily care required by patients in a given department [7]. The method was based on 278 nursing tasks listed and classified into three categories of care: 1) basic care (feeding, waste disposal, hygiene, clothing, comfort and mobility), 2) technical care (tasks related to medical diagnosis and therapy), 3) relational care (information and support to patients and families). Each care task was timed and classified into 4 groups corresponding to 4 levels of patient dependency, 4 levels of time required for the caregiver to perform these tasks. A time value of eight minutes and 20 s has been set per SIIPS point. Coefficients of 1, 4, 10 and 20 were established based on the intensity of care workload required for a patient. Nursing intensity per patient over 24 h could vary from three points (basic care = 1 + technical care = 1 + relational care = 1) if the patient was autonomous, to 60 points (basic care = 20 + technical care = 20 + relational care = 20) if the patient was totally dependent. As a result, time spent by caregiver could range from 25 to 500 min per patient per day. Nursing intensity was therefore scored by a global assessment, based on care required for a patient over the last 24 h. Technical and relational care was assessed by Nurses, and basic care was assessed by healthcare assistants, present in the morning before the afternoon shift. The choice of rating (1, 4, 10, 20) was based on three grids (See illustration, Additional file 1) that specify the level of intensity corresponding to care required for a patient in each of the three categories of care (basic care, technical care, relational care). A coefficient of nursing intensity per department was calculated (sum of scores divided by 60 and cumulative working hours). A coefficient between 0.6 and 1.0 was considered as normal workload, greater than 1.0 for the overload and less than 0.6 for the sub-activity.

**Fig. 1:**
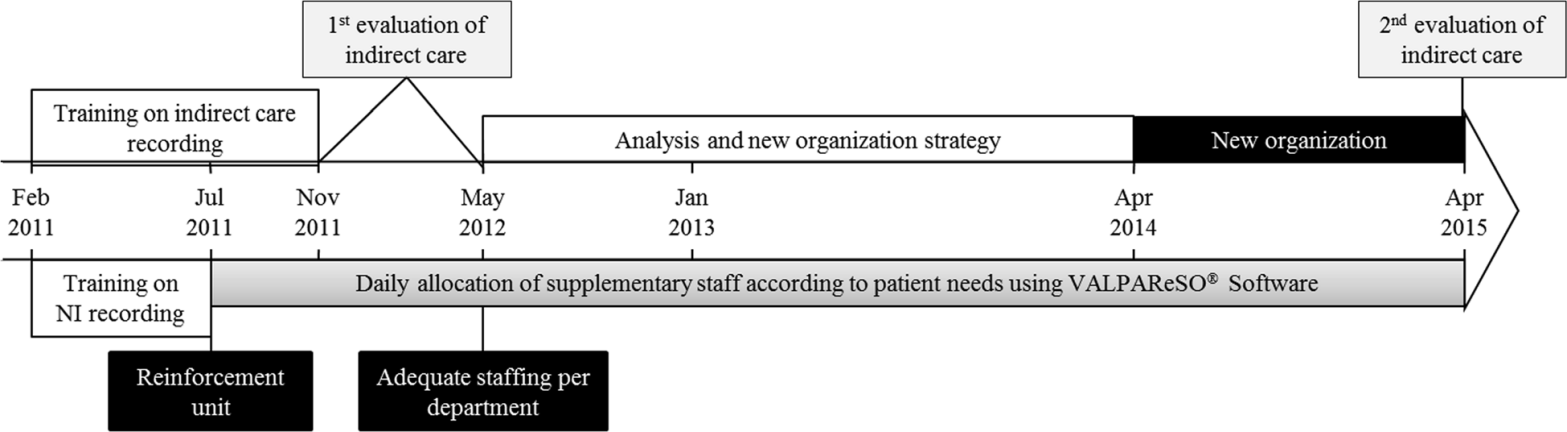
Course of the study. NI: nursing intensity

In addition to nursing intensity, healthcare provider workload was quantified through the evaluation of indirect care. These activities were directly linked to the organizational system of each department and correspond to tasks of catering, logistics, administrative and communication, guidance, training, and research (See form, Additional file 2). It refers to tasks that do not directly affect the patient (e.g., when nurses order medication and make examination appointments, or when healthcare workers order meals, ensure bio-cleaning of the environment near the patient, accompany a patient for an examination in another department, or when cleaners ensure bio-cleaning of the unit). The evaluation per department was carried out over seven successive working days (Fig. 1). The percentage of indirect care activity was calculated by dividing the number of hours spent on indirect care by the number of hours worked by healthcare workers in the unit with all staff present during a week.

The distinction between the two indicators is that nursing intensity includes care that directly affects the patient (e.g., giving a medication) while indirect care includes all tasks that do not directly affect the patient (e.g., ordering medication), the caregiver is not at his bedside.

### Satisfaction surveys

Patient and worker satisfaction surveys have been developed by the management in consultation with the unions (See documents, Additional files 3 and 4).

### Process

The process of the study was guided by the Ottawa model of research use [11].

#### Step 1: Ground preparation

In agreement with the Human Resources Department, the Healthcare Coordination Department has strengthened the replacement unit with available resources, using the budget previously allocated to interim and the additional staff allocated to the various units. This replacement unit may be called a “float pool” by other institutions, where professionals were assigned to other unit as needed.

Initial training on healthcare workload measurement tools was provided to managers, nurses and healthcare workers by an external organization (GRIEPS, training and consulting organization). Managers have been trained to become trainers. Their role was to ensure the quality and completeness of the data provided. A project team was formed and organized the implementation of nursing intensity and indirect care measurements to assess workload.

#### Step 2: Communication

A communication campaign was launched in February 2011 to inform all stakeholders in the institution about the implementation of the workload measurement tools and the strengthening of the reinforcement unit, which would be used accordingly.

#### Step 3: Assess obstacles and facilitators

A major difficulty was related to the production mode and use of the indicators. For instance, “to score” workload associated with patient care was not an intuitive method for the healthcare providers. An important phase of training and benchmarking took place at the beginning of the project. To overcome resistance to change, the pedagogical position chosen was based on two principles: 1) the similarity between the nursing intensity rating and the logical reasoning already mastered by caregivers, which consists in assessing patient clinical condition using validated professional scales (numerical rating scale); and 2) Simulations of scoring based on real clinical cases encountered in each department. At the same time, the VALPAReSO® software was made available to the care units to enable the daily recording of nursing intensity scores by caregivers. With this software, scoring only requires three minutes per day and per sector.

Moreover, to facilitate the logistical reorganization, providers were asked to reflect on organisational factors that needed to be improved. In each department and during dedicated work meetings, healthcare assistants thought together with the manager on reorganization to absorb tasks that were previously performed by cleaners due to task slippage (e.g. patient meal distribution), and which task to delegate to logistics professionals. Work meetings also happened between healthcare managers and managers from the supply centre, the pharmacy, the laundry department, the transport and the catering services.

#### Step 4: Select and monitor knowledge application strategies

A half-time healthcare manager provided quality controls, methodological support and covered tool operation. She had access to a dashboard summarizing the nursing intensity scores (See printscreen, Additional file 5), and used it as a decision-support tool in order to regulate workforces according to departments with the highest healthcare workload.

Regular quality controls by the healthcare managers guaranteed the relevance of ratings and compliance with the scoring method. Audits were carried out at least twice a year. They were conducted over one day and compared nursing intensity scoring with the content of patient records.

#### Step 5: Monitor the application of scoring

To be usable, the nursing intensity information had to be comprehensive (at least 90% of patients with a score on at least 90% of days per month). Each month, a bulletin was sent to healthcare managers with the scoring percentage of their department. Below 90%, the department could not benefit from reinforcement.

### Analysis

The primary outcome was the nursing intensity score, mirroring patient care needs, which was measured daily from July 2011 to December 2015. The average score per month and per department was used. The other main outcome was the time spent on indirect care by nurses and healthcare assistants, measured over 7 days in 2012 and in 2015. Indirect outcomes like patient and health providers satisfaction were also surveyed. Means +/− standard deviations or medians and ranges, counts, percentages and confidence intervals were used to describe continuous and categorical variables, respectively. Normal distributions were verified and T-test or Mann-Whitney tests were used to compare continuous data of independent samples where appropriate. Chi-square or Fisher tests were used for categorical variables. An alpha level of 0.05 was used for all statistical tests. XLSTAT 2015 (Addinsoft) was used for statistical analysis.

## Results

Nursing intensity was recorded every morning by nurses and healthcare assistants from July 2011 to December 2015, and replacement/supplementary staff were allocated on a daily basis (Fig. 1). Close to 1200 nurses (from 1132 to 1137 full time equivalent) and 1000 healthcare assistants (from 970 to 966 full time equivalent) were involved, and took care of around 75,000 patients each year. Nurse and healthcare assistant median ages were 40 years old and 80% were women.

### Nursing indicators

**Fig. 2:**
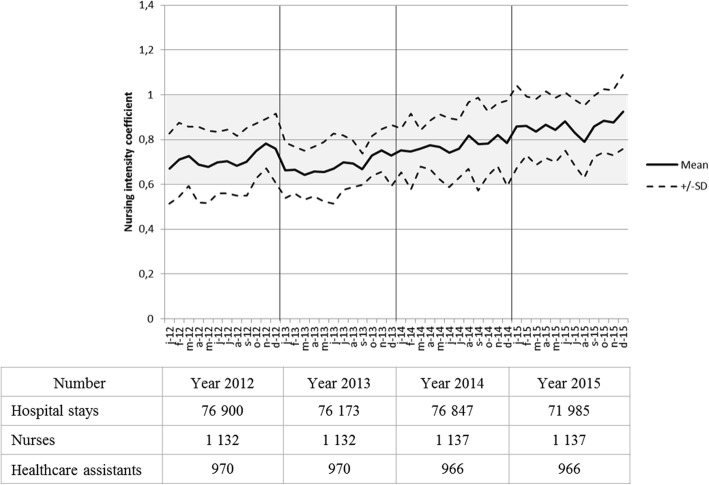
Nursing intensity coefficient, hospital stays and caregivers number. Coefficient measured per month from January 2012 to December 2015. Range of normal workload is highlighted in grey. SD: standard deviation. Numbers of nurses and healthcare assistants correspond to full time equivalent

From July 2011 to May 2012, the median of nursing intensity coefficient was 0.71 with a range from 0.47 to 0.90. Therefore, team composition per department was reassessed in order to balance the workload between all services. Four departments (geriatric short stay care, orthopaedic surgery, oncology and visceral surgery) were reinforced by one healthcare worker each. These units that received additional staff had a chronically high care load compared to other units. The result was a decreased disparity in nursing intensity between departments in 2013, which reach a median of 0.67 [0.54–0.78] in September 2013. In 2014, independently of the study, the health organization policy evolved towards the development of very short-term care. As a result, full inpatient beds were closed or converted to partial hospitalization beds. This is why the inpatient units, which previously housed a proportion of semi-autonomous patients and a proportion of highly dependent patients, have seen an increase in the proportion of dependent patients. These more dependent patients needed a higher level of care resulting in an amplified nursing intensity (Fig. 2). It had created new disparities between departments. In December 2015, the median of nursing intensity coefficient was 0.96 with a range from 0.46 to 1.21. These disparities were lessened by the daily use of the reinforcement unit.

Over the study period, all the departments adopted the nursing intensity scoring and its completion rate was 95%. Through the reinforcement unit, more than 86% of absent staff (training, sickness, maternity leave…) were replaced.

On a short term, i.e. on a daily basis, nursing intensity analysis allowed a rebalancing of human resources in hospitalisation departments by comparing the ratings of patient needs and allocating additional staff to the units with the highest care burden. The nursing intensity indicator became a sort of “standard metre”, a steering tool shared by the management team. Specific and shared vocabulary allowed for comparison between departments. In department with overload situation, scores per care were analysed. If basic care had the highest score, an additional healthcare assistant was assigned as reinforcement in the concerned department. If technical care had the highest score, an additional nurse was assigned.

Medium term, i.e. quarterly and annual, analysis of data allowed mapping of the burden of care and characterizing the various hospitalization units through department and patient profiles. It served as a basis for discussion and was a common steering tool.

In the context of restructuring departments, these data were used to model future (long term) organisations and determine staffing requirements in terms of qualifications and number of staff. Patient profiles and nursing intensity scores characterized a unit, and were used for negotiations in the allocation of resources, thus contributing to cost control.

### First evaluation campaign of indirect care

**Fig. 3:**
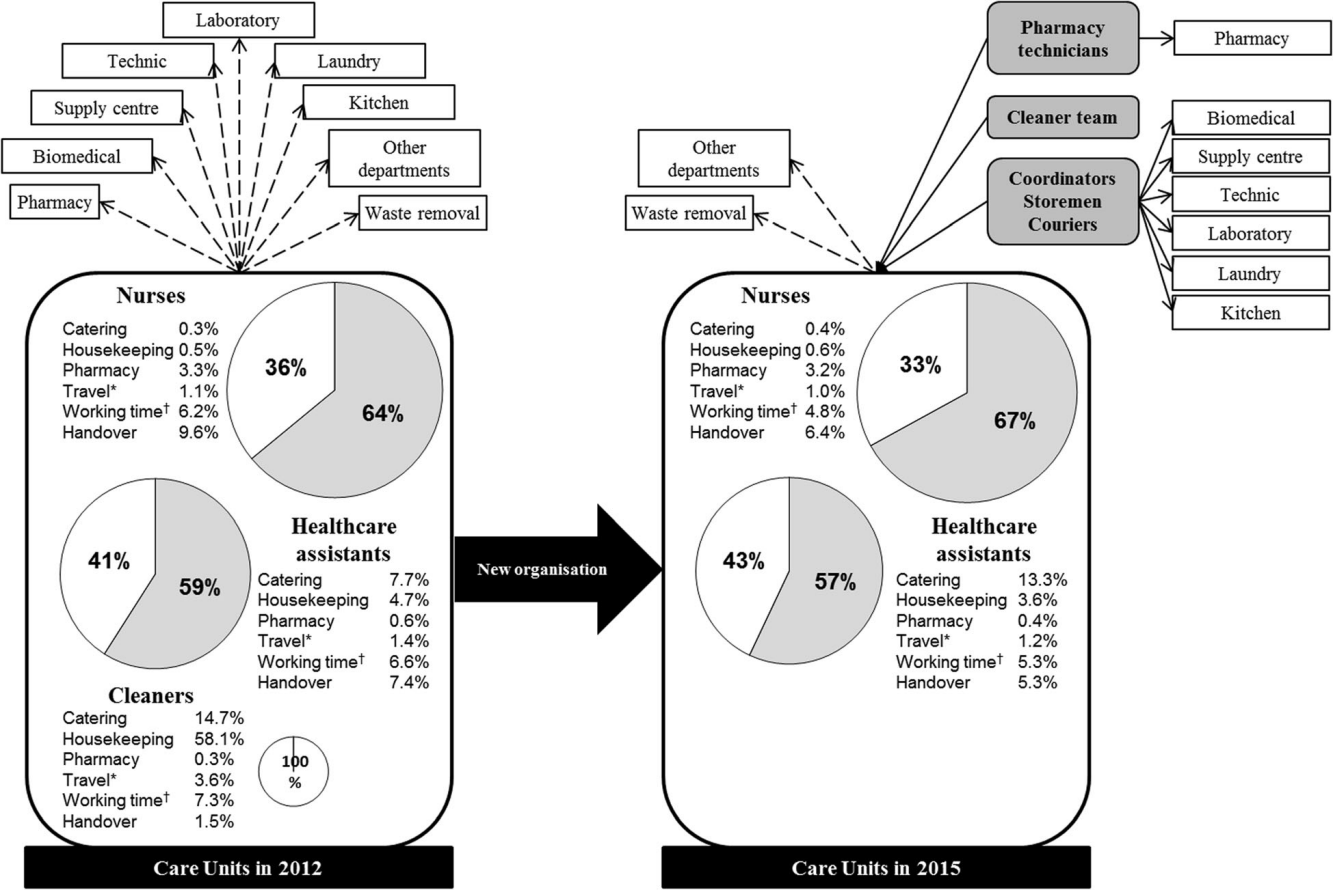
Care unit organisation in 2012 and 2015. Travel patterns to and fro the care units, and percentage of time spent on care (grey) and indirect care (white) by nurses, healthcare assistants and cleaners. Percentage of time spent on main revised tasks in the new organization strategy are detailed. New logistics professions are in dark grey. *Travel out of the care unit; ^†^ Management of working time

The 1st evaluation of indirect care was held between November 2011 and May 2012. All nurses, healthcare assistants and cleaners comprehensively filled out the indirect care form. During the campaign, 3927 patients were hospitalized in the departments. Globally, the indirect care represented 6144 h out of the 15,977 h of nurses and healthcare assistants working time (Table 1), i.e. 36.4 and 40.9% of time spent on indirect care by nurses and healthcare assistants, respectively (Fig. 3). Indirect care where hospital staff spent most of their time were cleaning, handover, catering, management of working time, delivery and stretcher, medication management, logistics and linen management, administrative tasks and hygiene. Disparities regarding time spent in management of working time, on the phone, and at administrative tasks related to patient medical record were observed between departments. Moreover, this study interestingly highlighted task slips between healthcare assistants and cleaners.

**Table 1:** Setting, participants and results of the two indirect care measurement campaigns

	2012	2015
Setting (Nb)		
Participating departments^a^	23	20
Hospitalized patients	3927	3738
Healthcare workers participating to indirect care recording (person-week)		
Nurses	1132	1137
Healthcare assistants	970	966
Cleaners	105	0
Time spent on indirect care (hours) over a week		
Nurses	3131	2895
Healthcare assistants	3013	3167
*Ratio (hours of indirect care/person)*		
Nurses	2,77	2,55
Healthcare assistants	3,11	3,28

^a^Differential between 2012 and 2015 are due to merged departments

### New organization strategy

Based on the indirect care activity results, the main organization revision was implemented in April 2014 and concerned five working areas: 1) time spent on handover, 2) working time management (some tasks performed during the day were transferred during the night), 3) connections between services and the pharmacy (nurses and healthcare assistant are no longer going to the pharmacy, but pharmacy technicians are now coming to the units), 4) housekeeping (creation of cleaner teams independent from care units), 5) food management (reorganization with the catering service, optimization of meal cart organisation, ask patients what they do not like instead of what they like) (Fig. 3).

The most efficient service in handover (i.e. intensive care unit) served as a model in order to define a common procedure. At first, written handover was read together by the newly arrived staff. Then, they met the departing staff for oral transmission and exchanged on specific points. Meetings, where teams from all units were brought together, were avoided. Healthcare managers from each department were in charge of implementing this harmonized model of handover.

In order to enable healthcare providers to focus on their healthcare tasks, pooled resources of new logistics professions were created and systematically used starting from April 2014. These logistics professionals included couriers and storemen (14.9 full time equivalents) who transported and stored materials, supplies, meals, and laboratory specimen, and coordinators (20.3 full time equivalents) who comprised formal healthcare assistants and ensured regular supply of the departments.

### Second evaluation campaign of indirect care

One year after the implementation of the new organization, in April 2015, nurses and healthcare assistants filled out again the indirect care activity form. Cleaners did not take part of this campaign as they were not any more part of a specific department. Over the study period, 3738 patients were taken care. Globally, the indirect care represented 6062 h out of the 15,985 h of working time (Table 1), i.e. 33.5 and 43.1% of time spent on indirect care by nurses and healthcare assistants, respectively (Fig. 3). The total indirect care activity ratio was reduced by 8% and increased by 5.5% for nurses and healthcare assistants, respectively. At department level, the average number of hours spent by a nurse on indirect care has significantly decreased from 3.0 (+/− 0.4) hours in 2012 to 2.7 (+/− 0.4) hours in 2015 (*p* = 0.041). Hence, nurses spared 18 min per working-day (341 h a week for the 20 departments) that were reinvested on patients ‘care. The average number of hours spent by a healthcare worker on indirect care increased slightly from 3.3 (+/− 0.5) in 2012 to 3.4 (+/− 0.5) in 2015, but this was not statistically significant (*p* = 0.441). This new campaign showed an improvement in time spent on handover. Healthcare providers left less frequently and spent less time outside their unit. However, the catering processes still need revision (Fig. 3).

### New organization indirect outcomes

As the reorganization provided more time for nurses at patient bedside and refocused healthcare workers on their own tasks, patient care should have improved and may have had an effect on patient satisfaction. According to the surveys made in 2012 and 2015, patient satisfaction has been maintained, and even seems to have improved concerning room comfort and information received at discharge (See tables, Additional file 6). Nurse and healthcare assistant perception of working conditions were well-preserved (See tables, Additional file 7). Indicators were experienced as an acknowledgment of healthcare workload by healthcare providers.

## Discussion

Nurses and healthcare assistants represent the largest component of the healthcare workforce and play a key role in patient safety and quality of care. Studies have shown that higher levels of nurse staffing are associated with lower rates of adverse outcomes, and with higher quality of care [12, 13]. However, Griffiths et al. [14] showed that the association between quality of care and organisation was much stronger than the association with nurse staffing level. Therefore, we aimed at both providing daily adequate staffing and improving organisational factors. To achieve these goals, we needed to objectify the subjectivity of workload in order to optimize healthcare human resources. In parallel, a diagnosis of our logistic organizations was necessary in order to define roles in a more consistent fashion, to identify all non-caregivers that could be entrusted, and thus to enable nurses to focus on healthcare provision.

Therefore, we chose and implemented nursing intensity recording commonly used for statistical purposes, and converted it into a dynamic management tool through the VALPAReSO® software. In parallel, a reinforcement unit was created and was constituted by healthcare assistants and highly qualified nurses. The objective was to continuously adapt human resources to the workload of patient-centred care as measured by the nursing intensity score. The culture of measuring the burden of care has become an integral part of caregiver daily lives. The availability, comparison and presentation of the results to the healthcare managers at department and unit level made cross-functional management possible. The results translated into significant elements for all professionals (intensity of basic, technical and relational care, patient profile, and care workload indicator) were shared and used as decision support tools.

Indirect care activities were measured using standards defined by the French Ministry of social affairs, health and city [9], and compared between departments. It enabled us to highlight weaknesses and strengths that led to revision of our healthcare processes. Not surprisingly, one of the main areas of work was handover, and specific efforts were dedicated to this task as it is an area of considerable vulnerability to patient safety [15]. In addition, several actions were taken in order to enable healthcare providers to focus on their healthcare tasks. A cross-functional reorganisation of internal supplier customers was initiated. This restructuring aimed at optimising healthcare and logistics activities, using constant resources by creating transversal teams and redeploying professionals. This approach was based on a collaborative management system that was open-ended between healthcare professionals and all providers. Beyond the objective of balancing the burden of care while controlling costs, our approach has included all producers of care and services in a process of harmonization and professionalization of practices based on a healthcare indicator recognized as reliable by all stakeholders.

Throughout the process of change, various difficulties were encountered. It was important to overcome barriers and to soften resistance to change in order to implement the new organisation and management strategy. Moreover, continuous improvement and organization review are necessary. Better collaboration between nurses and healthcare assistants is mandatory. Catering is still a consuming task that needs to be adjusted. Certain habits need to be revised in order to provide a more appropriate patient care, and to take into account daily life rhythm of patient.

The next steps are to extend the organization to all departments and to use healthcare workload indicators in relation with the diagnosis related groups. However, in the current state, scoring nursing intensity with SIISPS® is not adequate for psychiatry and obstetrics.

This was a single centre study and transfer of the methodology to other facility may need some adjustment as determining optimal staffing levels depend on a number of factors like facility size, dispersion across sites, and occupancy. In order to evaluate the reorganisation outcome, evolution of adverse events would have been an interesting indirect indicator about patient safety. Unfortunately, these data were not usable as the quality managers had heavily worked on the importance of reporting adverse events, and the increased number of recorded events was mostly due to the increased number of reports.

## Conclusion

The nursing indicator has become a daily management tool with transparent results for all departments. It enables the adjustment of available human resources by carrying out short-, medium- and long-term reflections, as close as possible to patient care needs in hospital units. Short term use of nursing intensity corresponds to its use on a daily basis for staff reinforcement. Medium term corresponds to quarter or annual report for mapping of burden of care per department. Long term assessment of nursing intensity was used for building predicting model of staff need in new or merged units.

## Additional files

Additional file 1: Scales used for nursing intensity assessment. (DOCX 67 kb)

Additional file 2: Indirect Care Form. (DOCX 56 kb)

Additional file 3: Patient satisfaction surveys. (DOCX 300 kb)

Additional file 4: Healthcare worker satisfaction surveys. (DOCX 298 kb)

Additional file 5: Example of nursing intensity coefficients from a screen shot of VALPAReSO Software dashboard. The colour code corresponds to the gradation of nursing intensity of the different departments. The red colour corresponds to the departments with the highest nursing intensity scores. The white colour corresponds to the departments with the lowest nursing intensity scores. Yellow, pale orange and dark orange correspond to in-between nursing intensity scores. (DOCX 293 kb)

Additional file 6: Results of Patient satisfaction surveys. (DOCX 62 kb)

Additional file 7: Results of Healthcare worker satisfaction surveys. (DOCX 54 kb)

## Data Availability

The datasets used and/or analysed during the current study are available from the corresponding author on reasonable request.

## Abbreviations

NICE: National Institute for Health and Care Excellence
PRN: Projet de recherche en nursing (Nursing research project)
SIIPS: Soins Infirmiers Individualisés à la Personne Soignée (Individualized Personal Care Nursing)
VALPAReSO: Valorisation paramédicale des soins (Paramedical value of care)
