# Supplementary material for: Revision of hospital work organization using nurse and healthcare assistant workload indicators as decision aid tools
Source: BMC Health Serv Res. 2019 Aug 7;19:554. doi: 10.1186/s12913-019-4376-7 (PMC6686463; doi:10.1186/s12913-019-4376-7)
Supplement: Supplementary file 1 — Scales used for nursing intensity assessment. (DOCX 67 kb) [file 12913_2019_4376_MOESM1_ESM.docx]

# Additional file 1

# Nursing indicator

**Basic care** (feeding, waste disposal, hygiene, dressing, comfort and locomotion)

| 1 |  |  | 4 |  |  |  |  |  | 10 |  |  |  |  |  |  |  |  |  | 20 |
| --- | --- | --- | --- | --- | --- | --- | --- | --- | --- | --- | --- | --- | --- | --- | --- | --- | --- | --- | --- |

| The patient is autonomous | The patient requires hardware support  (cane, basin, bowl…) | The patient requires physical assistance  (to wash his back, to sit in the chair …) | The patient is completely dependent;  Caregivers do in its place. |
| --- | --- | --- | --- |

**Technical care** (diagnostic and therapeutic procedures consecutive to medical prescription)

| 1 |  |  | 4 |  |  |  |  |  | 10 |  |  |  |  |  |  |  |  |  | 20 |
| --- | --- | --- | --- | --- | --- | --- | --- | --- | --- | --- | --- | --- | --- | --- | --- | --- | --- | --- | --- |

| 3 monitoring per day ;  light care  (ex: oral administration) | For situation requiring 6 monitoring per day  Requiring more care  (ex: drip) | For situation where more than 8 daily monitoring are needed or in situation of complex care.  (ex : blood transfusion) | For situation of critical failure, or requiring every hour monitoring.  (ex : central line) |
| --- | --- | --- | --- |

**Relation and educational care** (information and support to patients and families)

| 1 |  |  | 4 |  |  |  |  |  | 10 |  |  |  |  |  |  |  |  |  | 20 |
| --- | --- | --- | --- | --- | --- | --- | --- | --- | --- | --- | --- | --- | --- | --- | --- | --- | --- | --- | --- |

| Custom Information (reception, discharge ...) | Reformulation, explication | Educational approach, special situation  (following diagnosis announcement, opposition to care ...) | Support in crises |
| --- | --- | --- | --- |
|  |  | Quotations 10 to 20 highlight relational information written in patient record. | |
