# Supplementary material for: Revision of hospital work organization using nurse and healthcare assistant workload indicators as decision aid tools
Source: BMC Health Serv Res. 2019 Aug 7;19:554. doi: 10.1186/s12913-019-4376-7 (PMC6686463; doi:10.1186/s12913-019-4376-7)
Supplement: Supplementary file 2 — Indirect Care Form. (DOCX 56 kb) [file 12913_2019_4376_MOESM2_ESM.docx]

# Additional file 2

**Indirect care form**

**for healthcare workers**

**Department :**.....................................

**Date:**..................................................

**Nurse; Healthcare assistant; Cleaner (select your job)**

W**orkstation hours:**

Start :.........................................

End : ........................................

**How to fill out the form:**

Identify beside each activity, time or successive time devoted to this activity in minutes.

| Example: | **Total**  **Time (min)** |
| --- | --- |
| Hand washing, out of healthcare context...1 + 1 + 1 + 1........................... | **4** |

| **Hotel tasks** | Total |
| --- | --- |
| **1 - Catering** | Time (min) |
| - Order: meal forms (diet calculation)........................................…………….…………………………..  - Reception and control, including temperature monitoring ……………………………………………  - Meal tray preparation, including temperature monitoring……………………………………………..  - Meal preparation (baby bottle, gelled water, mash)…………………………………………………...  - Meal distribution ………………………………………………………………………………………  - Meal tray pickup and waste…………………………………………..………………………………..  - Dishes………………………………………………………………………………………………….  - Storage (groceries)……………………………………………………………………………………..  - Provision of water……………………………………………………………………………………... | -  -  -  -  -  -  -  - |
|  | T1 = |
| **2 – Linen management**  - Order…………………………………………………………………..……………………………….  - Reception et storage…………………………………………………………………..………………..  - Carriage preparation …………………………………………………………………..……………….  - Packaging of dirty laundry…………………………………………………………………..………… | -  -  -  - |
|  | T2 = |
| **3 - Housekeeping**  - Material preparation …………………………………………………………………………………...  - Cleaning :  • Daily room (soil et environment) ………………………………………………………………….  • End of stay (beds + environment) …………………………………………………………………  • Common parts ……………………………………………………………………………………  • Scouring ……………………………………………………………………………………………  • Moving furniture ………………………………………………………………………………….  • Bio cleaning traceability ………………………………………………………………………….. | -  -  -  -  -  - |
|  | T3 = |
| **4 – Waste management**  **-** Waste sorting.................................................................................................…………………………  - Waste packaging including transit to centralized storage............................………………………….. | -  - |
|  | T4 = |

| **Logistics** |  |
| --- | --- |
| **5 - Hygiene - Decontamination - Sterilization**  - Hand washing, out of healthcare context ……………………………………………………………...  - Cleaning-decontamination of:  • Instruments (or other equipment, incubator, bottle warmer)………………………………………..  • Work surface………………………………………………………………………………………...  - Cleaning (bedpan, bowl, commode chair) ……………………………………………………………  - Sterile equipment storage …………………………………………..………………………………….  - Preparation of decontamination solution ……………………………………………………………...  - Refrigerator storage + defrost (care room, office) + temperature monitoring………………………… | -  -  -  -  -  -  -  - |
|  | T5 = |
| **6 - Pharmacy**  **-** Order, receipt, control, storage (allocation) ….………………………………………………………..  - Order, receipt, control (emergency)…………………………………..………………………………..  - Preparation of daily medications ………………………………………..…………………………….  - Control of drug cabinet (stock - expiry) ……….………………………………..…………………….  - Narcotic Control ….………………….………………………………………………………………..  - Command, control, storage of narcotics ………………………………..……………………………..  - Order, receipt, control, storage of small equipment.………………..…………………………………. | -  -  -  -  -  -  - |
|  | T6 = |
| **7 - Laboratory**  - Order of sampling devices …………………………………………………………………………….  - Reception, control, storage of sampling devices ……………………………………………………...  - Preparation of tubes and exam forms ………………………………...……………………………….  - Collection and filling of results …………………………………… ………………………………… | -  -  -  - |
|  | T7 = |
| **8 - Management of equipment and supplies**  - Order, receipt, control, storage (allocation)…….…..……………….....................................................  - Reception, control……………………………………………………………………………………...  - Storage ………….………………......................................................................................  - Material distribution …………………………………………………………………………………..  - Control and maintenance of equipment (in unit, work forms) (checking ICU room, emergency trolley) …………………………………………………………………………………………………. | -  -  -  -  - |
|  | T8 = |
| **9 – Travel out of care unit toward**  - Kitchen………...............................................................…………………………………………….. - - Storage place of waste, linen....................................................................…………………………...  - Pharmacy……………………………………………………………………………..........................  - Operating room………………………………………………………………………………………  - Technical service…………………………………………………………………………………….  - Administrative department…………………………………………………………………………..  - Other care department……………………………………………………………………………….  - Hospital linen service…………………………………………………………………………..……  - Store, supply centre, stationery ……………………………………………………………………..  - Laboratory…………………………………………………………………………………………...  - Secretariat department……………………………………………………………………………….  - Radiology……………………………………………………………………………………………  - Admission desks…………………………………………………………………………………….  - Services for patients (mail, newspapers)……………………………………………………………..  - Travel with a patient (death chamber, smoking patient, stretcher)…………………………………...  - Photocopy……………………………………………………………………………………………. | -  -  -  -  -  -  -  -  -  -  -  -  -  -  -  - |
|  | T9 = |
| **10– Management of working time**  - Exchanges for schedule………………………………………………………………………………...  - Planning……..........................................................................................................................................  - Break (meal, coffee)……………………………………………………………………………………  - Cigarette break………...........................................................................................................................  - Surveillance (night)…………….....................................……............................................................... | -  -  -  -  - |
|  | T10 = |

|  |  |
| --- | --- |
| **Communication - Information - Relationship** |  |
| **11 - Handover**  - Written messages to inform other professionals (eg healthcare assistant, manager, physiotherapist...)  - Nursing intensity recording……………………………………………………………………………  - Oral handover...................................................................................…………..………………………  - Care planning change.............................………………………….…………………………………… | -  -  -  - |
|  | T11= |
| **12 - Collaboration with physicians**  - Medical examination………..........................................................………………………………….…  - Medical examination of new patient / emergency………........................................................………..  - Review meeting….............................................................………….…………………………………  - Find a physician……………………………………………………………………………………….. | -  -  -  - |
|  | T12 = |
| **13 – Various exchange**  - With visitors (not to be confused with relational or educational care with families)…………….........  - With other professionals……….......................................................……..............................................  - Other………………………………………….........................................…….........…………………. | -  -  - |
|  | T13 = |
| **14 – Phone**  **-** Refer to the daily record of phone calls ……........…………................................................................. | - |
|  | T14 = |
| **15 – Meeting**  **- Work meeting** ....................................................................................................………………...…… | - |
|  | T15 = |
| **Administrative tasks** |  |
| **16 - Management of patient flow**  - Patient flow recording ……………………………….……………………..........................................  - Formalities related to special cases (death, running away, protected adult...)…………………………  - Bed management (admission, discharge)…………………………………………………………….. | -  -  - |
|  | T16 = |
| **17 - Administrative procedures**  **-** Preparation and monitoring of patient records.......................................................................................  - Administrative tasks (appointment for consultation, imaging exploration)…………………………...  - Phone line opening…………………………………………………..…………………………………  - Various services (newspapers order – TV...) by phone …….……………………………………….  - Declaration of an adverse event ………………………………............................................................ | -  -  -  -  - |
|  | T17 = |
| **Coaching - Training - Research** |  |
| **18 - Students and staff**  - Welcome........................................................................................….......…………………..................  - Coaching............…………….................................................................................................................  - Appraisal………………………………………………………………………………………………. | -  -  - |
|  | T18 = |
| **19 - Training - Teaching and Research**  - Training report........................................................................…………...……………………………  - Educational monitoring...................................................................................………………………..  - Teaching.......................................................................……………………………………………….  - Reading.....................................................................................………………………………………  - Research......................................................................................……………………………………..  - OCRA recording……………………………………………………………………………………… | -  -  -  -  - |
|  | T19 = |
|  |  |

Specific comments:

| **Dressing - Undressing** | | |  |  |  |
| --- | --- | --- | --- | --- | --- |
| **20 –**Locker room.......................................................................................….......………………… | | | - |  |  |
|  | | | T20 = |  |  |
| Unit code:............…….  Department:..............……  ..............................……. | | **Other Care Related Activities (OCRA)**  **Study**  **--------------**  **Daily record of phone calls** | **(OCRA4)**  - **Date**:.................................... | | |

| **Who picks up**  **the phone?** | | **Call duration** including travel time  (code X if duration <  30 seconds) | | **Origin of the message** | **Recipient** | **Comments** |  |
| --- | --- | --- | --- | --- | --- | --- | --- |
| grade |  | Hour | Duration |  |  |  |  |
|  |  |  |  |  |  |  |  |
