# Supplementary material for: Revision of hospital work organization using nurse and healthcare assistant workload indicators as decision aid tools
Source: BMC Health Serv Res. 2019 Aug 7;19:554. doi: 10.1186/s12913-019-4376-7 (PMC6686463; doi:10.1186/s12913-019-4376-7)
Supplement: Supplementary file 3 — Patient satisfaction surveys. (DOCX 300 kb) [file 12913_2019_4376_MOESM3_ESM.docx]

# Additional file 3

*Year - Hospital department*

**Demographics**

Age:

- less than 15
- from 15 to 25
- from 26 to 35
- from 36 to 45
- from 46 to 55
- from 56 to 75
- 76 and more

Gender**:**

- Female
- Male

**Reception and condition of stay**

|  | very satisfied | Satisfied | Dissatisfied | Not satisfied |
| --- | --- | --- | --- | --- |
| Reception at care departments |  |  |  |  |
| Quiet room |  |  |  |  |
| Room comfort |  |  |  |  |
| Cleanliness of the room |  |  |  |  |
| Ambient room temperature |  |  |  |  |

**Catering services**

|  | very satisfied | Satisfied | Dissatisfied | Not satisfied |
| --- | --- | --- | --- | --- |
| Food temperature |  |  |  |  |

**Medical and paramedical care**

|  | very satisfied | Satisfied | Dissatisfied | Not satisfied |
| --- | --- | --- | --- | --- |
| Staff availability |  |  |  |  |
| Information received on care and health status |  |  |  |  |
| Information received about care and examinations |  |  |  |  |
| Respect of information confidentiality |  |  |  |  |
| Respect of privacy and dignity during care |  |  |  |  |
| Management of pain |  |  |  |  |
| Quality of care |  |  |  |  |

**Discharge**

|  | very satisfied | Satisfied | Dissatisfied | Not satisfied |
| --- | --- | --- | --- | --- |
| Hospital discharge organization |  |  |  |  |
| Information received at hospital discharge |  |  |  |  |

|  | very satisfied | Satisfied | Dissatisfied | Not satisfied |
| --- | --- | --- | --- | --- |
| **Overall satisfaction** |  |  |  |  |
