# Supplementary material for: Revision of hospital work organization using nurse and healthcare assistant workload indicators as decision aid tools
Source: BMC Health Serv Res. 2019 Aug 7;19:554. doi: 10.1186/s12913-019-4376-7 (PMC6686463; doi:10.1186/s12913-019-4376-7)
Supplement: Supplementary file 4 — Healthcare worker satisfaction surveys. (DOCX 298 kb) [file 12913_2019_4376_MOESM4_ESM.docx]

# Additional file 4

*Year - Hospital department*

**Demographics**

Age:

- less than 25
- from 25 to 34
- from 35 to 44
- from 45 to 54
- 55 and more

Gender**:**

- Female
- Male

**What is your status?**

- Official agent
- Permanent contract
- Fixed-term contract

**In which supra-department do you work?**

- Women and children
- Infectious diseases and cardiovascular diseases
- Surgery
- Neurology, Oncology, Pulmonary

**What is your occupation?**

- Nurse
- Healthcare assistant
- Cleaner

**Your working time and its organization**

|  | very satisfied | Satisfied | Dissatisfied | Not satisfied |
| --- | --- | --- | --- | --- |
| Are you satisfied with your working hours? |  |  |  |  |
| Is your work schedule respected? |  |  |  |  |
| In general, are you satisfied with the organization of your working time? |  |  |  |  |

**Your workstation**

|  | Yes, absolutely | Pretty much, yes | Rather no | Not at all |
| --- | --- | --- | --- | --- |
| Are you satisfied with your current position? |  |  |  |  |
| In your department, are the tasks clearly defined |  |  |  |  |
| Do you feel you are doing a useful job |  |  |  |  |

**Your working condition**

|  | very good | Pretty good | Rather bad | Very bad |
| --- | --- | --- | --- | --- |
| You would say that your relationships with your colleagues are |  |  |  |  |
| You would say that your relationships with your local manager are |  |  |  |  |
| The working atmosphere in your department is |  |  |  |  |

|  | Yes, absolutely | Pretty much, yes | Rather no | Not at all |
| --- | --- | --- | --- | --- |
| Do you feel safe in your workplace? |  |  |  |  |

|  | very satisfied | Satisfied | Dissatisfied | Not satisfied |
| --- | --- | --- | --- | --- |
| Overall, are you satisfied with the working conditions |  |  |  |  |
