# Supplementary figures and images for: Revision of hospital work organization using nurse and healthcare assistant workload indicators as decision aid tools
Source: BMC Health Serv Res. 2019 Aug 7;19:554. doi: 10.1186/s12913-019-4376-7 (PMC6686463; doi:10.1186/s12913-019-4376-7)

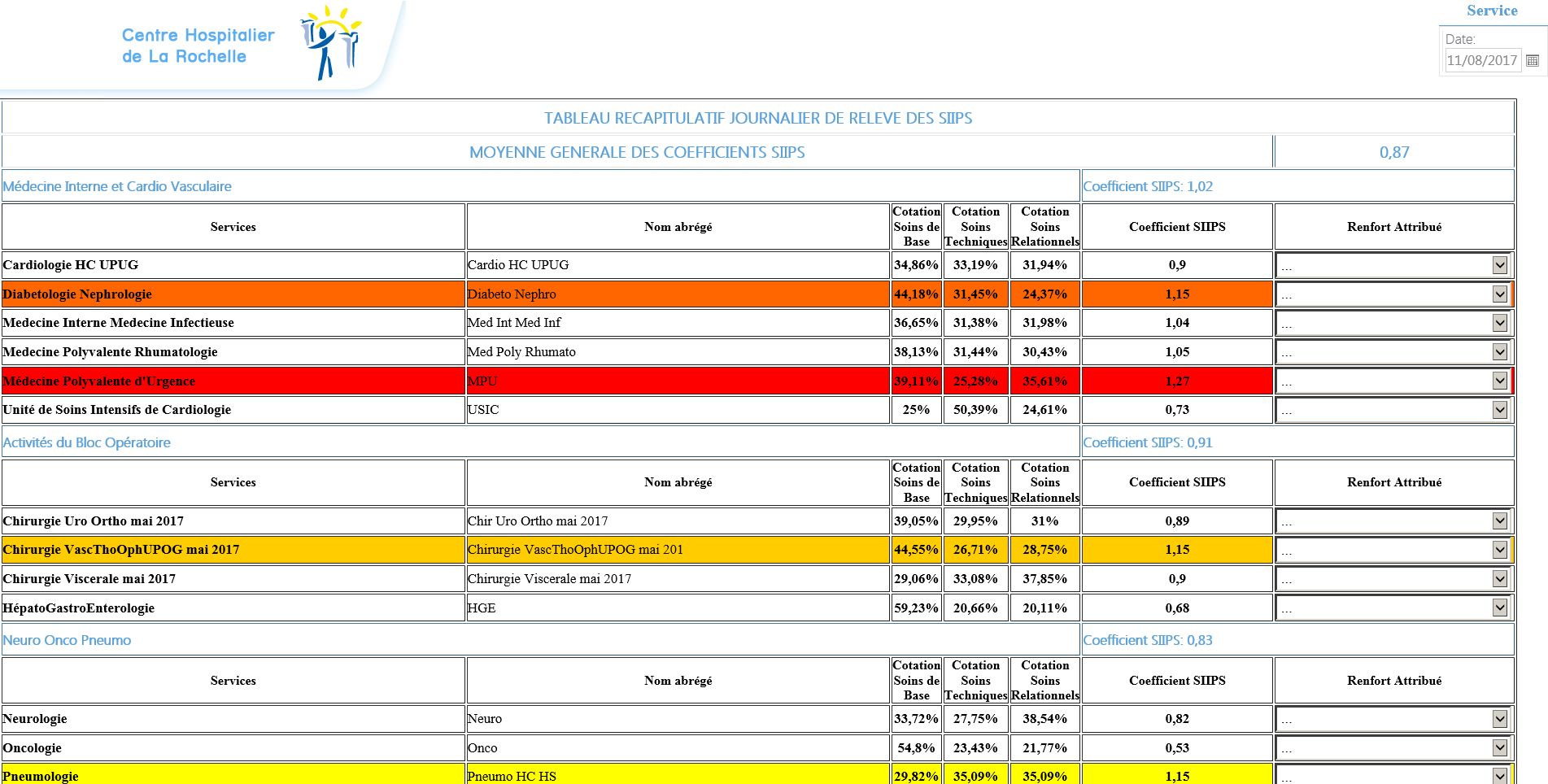

Supplement: Supplementary file 5 — Example of nursing intensity coefficients from a screen shot of VALPAReSO Software dashboard. The colour code corresponds to the gradation of nursing intensity of the different departments. The red colour corresponds to the departments with the highest nursing intensity scores. The white colour corresponds to the departments with the lowest nursing intensity scores. Yellow, pale orange and dark orange correspond to in-between nursing intensity scores. (DOCX 293 kb) [file 12913_2019_4376_MOESM5_ESM.docx]
