# Supplementary material for: Revision of hospital work organization using nurse and healthcare assistant workload indicators as decision aid tools
Source: BMC Health Serv Res. 2019 Aug 7;19:554. doi: 10.1186/s12913-019-4376-7 (PMC6686463; doi:10.1186/s12913-019-4376-7)
Supplement: Supplementary file 6 — Results of Patient satisfaction surveys. (DOCX 62 kb) [file 12913_2019_4376_MOESM6_ESM.docx]

# Additional file 6

| **Departments** | **2012** | **2015** |
| --- | --- | --- |
| *Women and children:* Gynaecology, Neonatology, Paediatrics | 242 | 183 |
| *Infectious diseases and CVD:* Cardiac intensive care, Cardiology, Diabetes, Inpatient cardiology, Internal medicine, Medicine, Rheumatology | 1327 | 1171 |
| *Surgery*: Gastroenterology, Orthopaedics, Urology, Vascular and thoracic surgery, Visceral surgery | 1071 | 607 |
| Neurology, Oncology, Pulmonary department | 548 | 352 |
| **Total** | **3188** | **2313** |

Table 1: Number of patients who answered to satisfaction surveys led in 2012 and 2015. CVD: cardiovascular disease.

|  |  | **2012** | | **2015** | |  |
| --- | --- | --- | --- | --- | --- | --- |
|  |  | **n** | **(%)** | **n** | **(%)** | ***p*** |
| Reception at care departments | Dissatisfied | 41 | (1.3%) | 29 | (1.3%) | 1.000 |
|  | Satisfied | 3147 | (98.7%) | 2284 | (98.7%) |  |
| Quiet room | Dissatisfied | 410 | (12.9%) | 264 | (11.4%) | 0.113 |
|  | Satisfied | 2778 | (87.1%) | 2049 | (88.6%) |  |
| Room comfort | Dissatisfied | 212 | (6.6%) | 118 | (5.1%) | 0.018 |
|  | Satisfied | 2976 | (93.4%) | 2195 | (94.9%) |  |
| Cleanliness of the room | Dissatisfied | 96 | (3.0%) | 78 | (3.4%) | 0.482 |
|  | Satisfied | 3092 | (97.0%) | 2235 | (96.6%) |  |
| Ambient room temperature | Dissatisfied | 341 | (10.7%) | 225 | (9.7%) | 0.261 |
|  | Satisfied | 2847 | (89.3%) | 2088 | (90.3%) |  |
| Food temperature | Dissatisfied | 199 | (6.2%) | 167 | (7.2%) | 0.154 |
|  | Satisfied | 2989 | (93.8%) | 2146 | (92.8%) |  |
| Staff availability | Dissatisfied | 38 | (1.1%) | 41 | (1.8%) | 0.085 |
|  | Satisfied | 3150 | (98.9%) | 2272 | (98.2%) |  |
| Information received on care and health status | Dissatisfied | 174 | (5.5%) | 121 | (5.2%) | 0.762 |
|  | Satisfied | 3014 | (94.5%) | 2192 | (94.8%) |  |
| Information received about care and examinations | Dissatisfied | 150 | (4.7%) | 108 | (4.7%) | 1.000 |
|  | Satisfied | 3038 | (95.3%) | 2205 | (95.3%) |  |
| Respect of information confidentiality | Dissatisfied | 45 | (1.4%) | 35 | (1.5%) | 0.820 |
|  | Satisfied | 3143 | (98.6%) | 2278 | (98.5%) |  |
| Respect of privacy and dignity during care | Dissatisfied | 52 | (1.6%) | 35 | (1.5%) | 0.744 |
|  | Satisfied | 3136 | (98.4%) | 2278 | (98.5%) |  |
| Management of pain | Dissatisfied | 114 | (3.6%) | 88 | (3.8%) | 0.663 |
|  | Satisfied | 3074 | (96.4%) | 2225 | (96.2%) |  |
| Quality of care | Dissatisfied | 38 | (1.2%) | 31 | (1.3%) | 0.626 |
|  | Satisfied | 3150 | (98.8%) | 2282 | (98.7%) |  |
| Hospital discharge organization | Dissatisfied | 158 | (5.0%) | 112 | (4.8%) | 0.899 |
|  | Satisfied | 3030 | (95.0%) | 2201 | (95.2%) |  |
| Information received at hospital discharge | Dissatisfied | 123 | (3.9%) | 61 | (2.6%) | 0.016 |
|  | Satisfied | 3065 | (96.1%) | 2252 | (97.4%) |  |
| Overall satisfaction | Dissatisfied | 68 | (2.1%) | 57 | (2.5%) | 0.411 |
|  | Satisfied | 3120 | (97.9%) | 2256 | (97.5%) |  |

Table 2 : patient satisfaction survey results
