# Supplementary material for: Revision of hospital work organization using nurse and healthcare assistant workload indicators as decision aid tools
Source: BMC Health Serv Res. 2019 Aug 7;19:554. doi: 10.1186/s12913-019-4376-7 (PMC6686463; doi:10.1186/s12913-019-4376-7)
Supplement: Supplementary file 7 — Results of Healthcare worker satisfaction surveys. (DOCX 54 kb) [file 12913_2019_4376_MOESM7_ESM.docx]

# Additional file 7

|  | 2011 | | 2015 | |
| --- | --- | --- | --- | --- |
|  | (n=202) | | (n=257) | |
|  | n | (%) | n | (%) |
| **Sex** |  |  |  |  |
| Male | 9 | (4%) | 12 | (5%) |
| Female | 193 | (96%) | 243 | (95%) |
| **Age** |  |  |  |  |
| < 25 | 20 | (10%) | 11 | (4%) |
| 25-34 | 83 | (41%) | 117 | (45%) |
| 35-44 | 44 | (22%) | 69 | (27%) |
| 45-54 | 39 | (19%) | 42 | (16%) |
| > 54 | 16 | (8%) | 16 | (6%) |
| **Status** |  |  |  |  |
| Official agent | 157 | (78%) | 207 | (81%) |
| Permanent contract | 40 | (20%) | 36 | (14%) |
| Fixed-term contract | 4 | (2%) | 11 | (4%) |
| **Profession** |  |  |  |  |
| Nurse | 93 | (46%) | 144 | (56%) |
| Healthcare assistant | 83 | (41%) | 105 | (41%) |
| Cleaner | 26 | (13%) | 8 | (3%) |

Table S1: characteristics of healthcare workers who responded to satisfaction surveys led in 2012 and 2015

|  |  | **2012** | | **2015** | |  |
| --- | --- | --- | --- | --- | --- | --- |
|  |  | **n** | **(%)** | **n** | **(%)** | ***p*** |
| Work timetable | Dissatisfied | 38 | (19%) | 41 | (16%) | 0.482 |
|  | Satisfied | 160 | (81%) | 212 | (84%) |  |
| Schedule | Dissatisfied | 37 | (19%) | 46 | (18%) | 0.976 |
|  | Satisfied | 162 | (81%) | 206 | (82%) |  |
| Overall work organization | Dissatisfied | 24 | (12%) | 37 | (15%) | 0.580 |
|  | Satisfied | 171 | (88%) | 217 | (85%) |  |
| Current position | Dissatisfied | 30 | (15%) | 26 | (10%) | 0.164 |
|  | Satisfied | 166 | (85%) | 223 | (90%) |  |
| Relationship with colleagues | Dissatisfied | 2 | (1%) | 2 | (1%) | 1.000 |
|  | Satisfied | 198 | (99%) | 248 | (99%) |  |
| Relationship with the manager | Dissatisfied | 3 | (2%) | 5 | (2%) | 1.000 |
|  | Satisfied | 196 | (98%) | 244 | (98%) |  |
| Overall working conditions | Dissatisfied | 48 | (24%) | 57 | (24%) | 0.946 |
|  | Satisfied | 148 | (76%) | 183 | (76%) |  |
| Clear-cut tasks | Disagree | 15 | (8%) | 32 | (13%) | 0.129 |
|  | Agree | 181 | (92%) | 224 | (88%) |  |
| Feeling of doing useful work | Disagree | 11 | (6%) | 7 | (3%) | 0.222 |
|  | Agree | 189 | (95%) | 244 | (97%) |  |
| Feeling safe | Disagree | 26 | (13%) | 40 | (16%) | 0.487 |
|  | Agree | 171 | (87%) | 210 | (84%) |  |
| Work atmosphere | Poor | 15 | (8%) | 23 | (9%) | 0.610 |
|  | Good | 184 | (92%) | 228 | (91%) |  |

Table S2 : healthcare workers’ satisfaction survey results
